# Constructing an Isogenic 3D Human Nephrogenic Progenitor Cell Model Composed of Endothelial, Mesenchymal, and SIX2-Positive Renal Progenitor Cells

**DOI:** 10.1155/2019/3298432

**Published:** 2019-05-02

**Authors:** Lisa Nguyen, Lucas-Sebastian Spitzhorn, James Adjaye

**Affiliations:** Institute for Stem Cell Research and Regenerative Medicine, University Hospital Duesseldorf, 40225 Duesseldorf, Germany

## Abstract

Urine has become the source of choice for noninvasive renal epithelial cells and renal stem cells which can be used for generating induced pluripotent stem cells. The aim of this study was to generate a 3D nephrogenic progenitor cell model composed of three distinct cell types—urine-derived SIX2-positive renal progenitor cells, iPSC-derived mesenchymal stem cells, and iPSC-derived endothelial cells originating from the same individual. Characterization of the generated mesenchymal stem cells revealed plastic adherent growth and a trilineage differentiation potential to adipocytes, chondrocytes, and osteoblasts. Furthermore, these cells express the typical MSC markers CD73, CD90, and CD105. The induced endothelial cells express the endothelial cell surface marker CD31. Upon combination of urine-derived renal progenitor cells, induced mesenchymal stem cells, and induced endothelial cells at a set ratio, the cells self-condensed into three-dimensional nephrogenic progenitor cells which we refer to as 3D-NPCs. Immunofluorescence-based stainings of sectioned 3D-NPCs revealed cells expressing the renal progenitor cell markers (SIX2 and PAX8), podocyte markers (Nephrin and Podocin), the endothelial marker (CD31), and mesenchymal markers (Vimentin and PDGFR-*β*). These 3D-NPCs share kidney progenitor characteristics and thus the potential to differentiate into podocytes and proximal and distal tubules. As urine-derived renal progenitor cells can be easily obtained from cells shed into urine, the generation of 3D-NPCs directly from renal progenitor cells instead of pluripotent stem cells or kidney biopsies holds a great potential for the use in nephrotoxicity tests, drug screening, modelling nephrogenesis and diseases.

## 1. Introduction

Many disease conditions, including renal diseases, require replacement of tissues or organs. Organ or tissue transplantation is the only effective and most widely used medical treatment [1]. As stem cells can be used for the generation of autologous, specialized cell types, stem cell-based therapies are an alternative to transplantation [2]. However, both treatments face major problems: worldwide donor shortage, poor immunohistocompatibility between the donor and recipient, and the probability of side effects such as teratoma and tumor formation upon stem cell therapy. An alternative to kidney transplantations is the use of renal progenitor cells, which can be isolated from human urine [1], in order to generate kidney cell types and subsequently transplantable renal tissues. Physiological processes in the kidney result in thousands of viable kidney cells being shed into the urine [1, 3]. The cell type of interest, i.e., urine stem cell or urine-derived renal progenitor cell (UdRPC), is required for the renewal of kidney cells [4]. UdRPCs have a rice grain-like morphology [3] and share stem cell characteristics including clonogenicity, high expansion capacity, multipotent differentiation potency, and self-renewal driven by SIX2 [3, 5, 6]. In addition, these cells have the potential to be differentiated into numerous cell types present within the kidney.

The three-dimensional organoid technology is another alternative. Here, cells of the organ of interest, such as heart, liver, or kidney, are generated from induced pluripotent stem cells (iPSCs) in a 3D manner, named organoids. Because these organoids are composed of organ-specific cells which can self-organize, they are able to recapitulate some of the typical organ structures and functions [7, 8]. Other three-dimensional models include gastruloids, defined as *in vitro* multicellular models capable of mimicking the gastrulation process [9]. Published reports have shown successful generation of organoids derived from tissues such as the optic cup [10], hypophysis epithelium [11], intestine [12], cerebrum [13], and kidney [14]. Current shortfalls of existing organoid models include the lack of vascularization and the associated supply with nutrients and oxygen through blood flow as well as the organization of complex structures. Moreover, this kind of tissue engineering is based on the use of specific inducing factors and scaffolds, which cannot fully recapitulate the *in vivo* microenvironment needed for cell-cell interactions in the changing fluidity during organogenesis [15]. In light of these shortfalls, the generation of organoids by imitating the multicellular interactions in the *in vivo* organ is the next step needed to enhance organoid technology, especially in the kidney.

Here, we describe the generation and characterization of 3D-NPCs (three-dimensional nephron progenitor cells) composed of three cell types—SIX2-positive urine-derived renal progenitor cells (UdRPCs), UdRPC-iPSC-derived mesenchymal stem cells (UdRPC-iMSCs), and endothelial cells (UdRPC-iECs) to mimic the multicellular organization of the *in vivo* organ. The combination of the aforementioned cell types resulted in self-condensed 3D-NPCs, maintaining the expression of the renal progenitor marker SIX2 when cultured in self-renewal supportive medium. 3D-NPCs can be harnessed for efficient generation of kidney organoids useful as a platform for studying nephrogenesis, kidney disease modelling, and nephrotoxicity testing.

## 2. Materials and Methods

### 2.1. iPSCs from Urine-Derived Renal Progenitor Cells (UdRPCs)

The iPSC line used, ISRM-UM51, here called UdRPC-iPSCs, was reprogrammed from renal progenitor cells (UdRPCs) isolated from urine samples as described before [16, 17]. ISRM-UM51 is of known HLA and has a CYP2D6 status of an intermediate metabolizer [17].

### 2.2. Differentiation of UdRPC-iPSCs to Endothelial Cells (UdRPC-iECs)

Prior to differentiation, UdRPC-iPS cells were adapted to E8 medium (STEMCELL Technologies) on Matrigel-coated plates (Corning Incorporated, #354277). At 80–90% confluency, cells were dissociated with 0.05% EDTA/PBS and single cells were seeded on Matrigel-coated plates with an addition of ROCK inhibitor Y-27632 (10 *μ*M) (Tocris Bioscience, #1254/1) for the first 24 h to improve cell survival. When the cell density reached 70-80%, mesoderm formation was induced for 44 h by cultivating the cells in E8 medium containing 25 ng/ml activin A (PeproTech, #120-14E), 5 ng/ml BMP4 (PeproTech, #120-05ET), and 1 *μ*M CHIR99021 (Tocris Bioscience, #TB4423-GMP/10) [18]. The medium was changed to E7 medium (STEMCELL Technologies, #05910) supplemented with 50 ng/ml BMP4, 5 *μ*M SB431542 (Tocris Bioscience, #1614), and 50 ng/ml VEGF-A (PeprotTech, #100-200) for three to five days. Endothelial cells were maintained in E7 medium supplemented with 50 ng/ml VEGF-A or Medium 200 (Gibco, #M200500) at 37°C and 5% CO_2_. Human umbilical cord vein endothelial cells (HUVECs) were used as a control.

### 2.3. Differentiation of UdRPC-iPSCs to Mesenchymal Stem Cells (UdRPC-iMSCs)

The UdRPC-iPSCs were split into single cells at a confluency of 90-100% by incubating with TrypLE (Gibco, #12604021) for 4 min. Single cells were seeded on Matrigel-coated 6-well plates. As described before, differentiation was prepared at 60-70% confluency [19, 20]. Maintenance medium was replaced with mesenchymal stem cell (MSC) differentiation medium composed of Minimum Essential Medium Eagle (*α*-MEM) (Sigma-Aldrich, #M8042-6x500ml), 10% FBS (Gibco, #10500064), 1% P/S (Invitrogen, #15140122), 1% GlutaMAX (Gibco, #35050061), and 10 *μ*M of the TGF*β*-receptor inhibitor SB431542. Cell differentiation was carried out for 14 days, and medium was changed every second day. Afterwards, the cells were passaged with TrypLE and were plated onto uncoated flasks. Passaging was continued until the cells attained an MSC-like morphology. The cells were kept in MSC cultivation medium (*α*-MEM, 10% FBS, 1% P/S, and 1% GlutaMAX) lacking SB431542. Differentiation of resulting UdRPC-iMSCs was carried out afterwards to evaluate their trilineage differentiation potential. In addition, the expression of typical MSC cell surface markers and the absence of hematopoietic markers were analysed via flow cytometry.

### 2.4. *In Vitro* Differentiation Assays

#### 2.4.1. Adipogenesis

Induction of adipogenesis was performed by incubating UdRPC-iMSCs in adipoinductive medium (Gibco, #A1007001) for three weeks with medium changes every second day. Formation of lipid droplets was detected via Oil Red O staining (Sigma-Aldrich, #1320-06-5).

#### 2.4.2. Chrondrogenesis

Chondrogenesis of UdRPC-iMSCs was induced with chondroinductive medium (Gibco, #A1007101), and cells were cultivated for three weeks with regular medium changes every second day. Cartilage formation was confirmed with Alcian Blue staining (Sigma-Aldrich, #33864-99-2).

#### 2.4.3. Osteogenesis

UdRPC-iMSCs were seeded in two wells of a 24-well plate and were incubated in osteoinductive medium (Gibco, #A1007201) for three weeks with medium changes every second day. To demonstrate the successful differentiation, calcium depots were identified with Alizarin Red staining (Sigma-Aldrich, #130-22-3).

### 2.5. Immunophenotyping of UdRPC-iMSCs

For the immunophenotyping, two biological replicates per cell type, namely, UdRPC-iMSCs, native UdRPCs and native human fetal MSCs [21], were analysed. Each replicate was divided into two aliquots, each containing 1 × 10^5^ cells. MSC phenotyping cocktail (cocktail of fluorochrome-conjugated monoclonal antibodies: CD14-PerCP, CD20-PerCP, CD34-PerCP, CD45-PerCP, CD73-APC, CD90-FITC, and CD105-PE) or the isotype control cocktail (cocktail of fluorochrome-conjugated monoclonal antibodies: mouse IgG1-FITC, mouse IgG1-PE, mouse IgG1-APC, mouse IgG1-PerCP, and mouse IgG2a-PerCP) was added to the samples. The cells were incubated with the respective antibody cocktail for 10 min at 4°C in the dark with occasional swaying of the tubes. Cells were washed afterwards, and the fixed samples were measured using the CyAn ADP (Beckman Coulter, CA, USA) and analysed using the Summit 4.3 software.

### 2.6. Immunofluorescence-Based Staining

Paraformaldehyde (Polysciences, #18814-10) fixed samples were washed with 1% Triton X-100/PBS (Merck, #9002-93-1). If staining for cell surface markers was intended, washing was done with PBS instead. After this step, samples were washed twice with PBS. To block unspecific binding sites, the sample was incubated with blocking buffer for 2 h at room temperature.

The primary antibody was incubated overnight at 4°C. The respective antibody was diluted following the instructions in Table 1. The following day, samples were washed three times with 0.05% Tween/PBS (Merck, #9005-64-5). The secondary antibody (solved 1 : 500 in blocking buffer/PBS of a ratio 1 : 2) and Hoechst (Thermo Fisher, #H3570) (1 : 5000) were added and incubated for 1 h in the dark at room temperature. After washing the samples twice with 0.05% Tween/PBS, the plates were kept in 1% PS/PBS at 4°C until evaluation under a fluorescence microscope X-Cite series 120 Lumen Dynamics (Zeiss).

### 2.7. Generation of 3D-NPCs Based on the Coculture of UdRPCs, UdRPC-iMSCs, and UdRPC-iECs

The medium for 3D-NPC maintenance was prepared by adding 5 ng/ml VEGF-A, 1 *μ*g/ml heparin, and 5 ng/ml EGF (PeproTech, #100-47) to renal progenitor maintenance medium (RPMM) [16, 17]. Confluent wells of UdRPCs, UdRPC-iMSCs, and UdRPC-iECs were incubated with TrypLE at 37°C until cells detached; thereafter, RPMM was added to stop the enzymatic reaction. UdRPCs and UdRPC-iMSCs were centrifuged at 250 ×g for 5 min, and UdRPC-iECs were centrifuged at 150 ×g for 5 min. After aspirating the supernatant and replenishing with fresh medium, cells were counted. The seeding ratio between the three cell types was 10 : 7 : 2 (UdRPCs, UdRPC-iMSCs, and UdRPC-iECs). The required cell number of one combination process was as follows: 1 × 10^6^ UdRPCs, 0.7 × 10^6^ UdRPC-iMSCs, and 0.2 × 10^6^ UdRPC-iECs. The cell types were resuspended in 1 ml RPMM. After mixing the three cell types, the cell suspension was added to a T25 flask with 7 ml RPMM, filling up to a total volume of 10 ml. ROCK inhibitor Y-27632 (10 *μ*M) was added on day one to ensure cell survival. The flask was placed in an upright position in the incubator at 37°C and 5% CO_2_. After 14 days of cultivation, condensed 3D-NPCs were transferred into a petri dish and kept at 37°C and 5% CO_2_ in a rotating incubator. Approximately 90% of the condensation experiments resulted in three-dimensional, nonadherent 3D-NPCs.

## 3. Results and Discussion

### 3.1. Derivation of UdRPC-iMSCs from UdRPC-iPSCs

**Figure 1:**
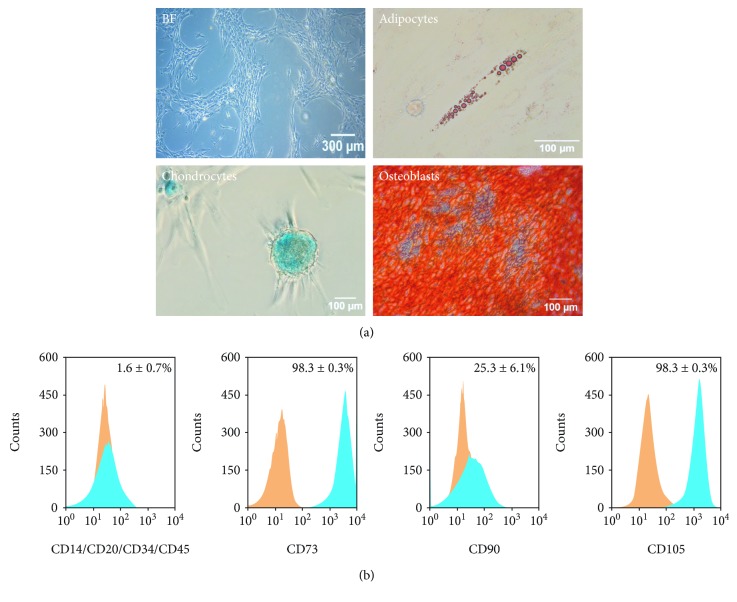
UdRPC-iMSCs are MSCs and bear characteristic MSC features. (a) Cell morphology and trilineage differentiation potential of UdRPC-iMSCs. UdRPC-iMSCs are elongated and spindle-shaped and possess trilineage differentiation potential to adipocytes, chondrocytes, and osteoblasts. (b) Immunophenotype of the generated UdRPC-iMSC line. Expression of MSC cell surface markers CD73, CD90, and CD105 and the hematopoietic markers CD14, CD20, CD34 and CD45 was analysed. Histograms of IgG control are displayed in orange, and histograms of MSC markers are displayed in blue (*n* = 2).

In this study, UdRPC-iMSCs were successfully generated from the iPSC line UM51 reprogrammed from UdRPCs [17]. The criteria defining mesenchymal stem cells include plastic adherence, trilineage differentiation potential to adipocytes, chondrocytes, and osteoblasts, expression of cell surface markers CD73, CD90, and CD105 (95% and higher), and absence of hematopoietic markers CD14, CD20, CD34, and CD45 [22]. The UdRPC-iMSCs displayed a fibroblast-like and spindle-shaped morphology and were able to adhere to plastic surfaces (Figure 1(a)). Their potential to differentiate to clinical relevant chondrogenic and osteogenic fate was observed by Alcian Blue and Alizarin Red staining (Figure 1(a)). Additionally, the potential to differentiate into adipocytes was shown by Oil Red O staining (Figure 1(a)).

Immunophenotyping of the UdRPC-iMSCs confirmed the expression of the typical MSC cell surface markers CD73, CD90, and CD105 and absence of the hematopoietic markers CD14/CD20/CD34/CD45 (1.59 ± 0.7%, Figure 1(b)). The levels of CD73 and CD105 were 98.30 ± 0.3% and 98.27 ± 0.3%, respectively (Figure 1(b)). UdRPC-iMSCs had a lower level CD90 (25.25 ± 6.1%) compared to bone marrow MSCs (Figure 1(b)). The reference gold standard bone marrow-derived MSCs have more than 95% CD90^+^ cells (Figure S1A). However, MSCs isolated from distinct organs and origins are known to express a diverse set of MSC cell surface markers and even with varying degrees of expression [23]. In contrast, the native UdRPCs, from which the UdRPC-iMSCs originate, have high levels of CD73 (99.11 ± 0.3%) and CD90 (79.28 ± 3.6%) and a low level of CD105 (10.92 ± 0.6%) (Figure S1B). Urine-derived stem cells have been described to express high levels of CD29, CD44, and CD73 (>98%) and a variable expression of CD54, CD90, CD105, and CD166 [24, 25]. These variations between MSCs may be due to inherent functional differences and the fact that the cells are part of a heterogeneous subpopulation within tissues [23]. Since UdRPC-iMSCs bear MSC features other than 95% CD90 expression, i.e., plastic adherence and the trilineage differentiation to adipogenic, chondrogenic, and osteogenic fate, UdRPC-iMSCs are considered MSC-like.

**Figure 2:**
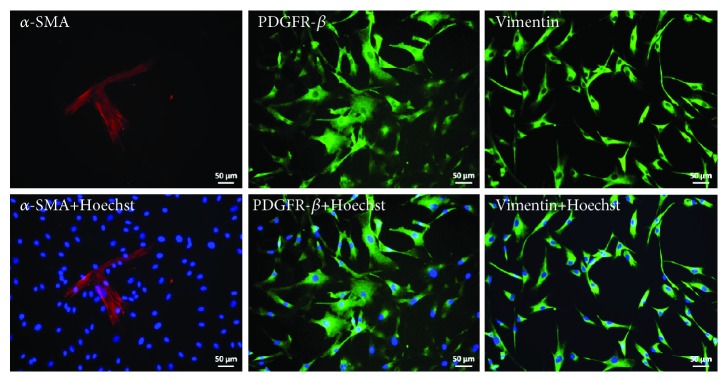
Expression of MSC markers in UdRPC-iMSCs. Stainings were carried out for the expression of the mesenchymal markers—*α*-SMA, Vimentin, and PDGFR-*β*. Cell nuclei were stained with Hoechst. Pictures were taken under 20x magnification.

Additionally, immunofluorescence-based staining also revealed the expression of the MSC markers *α*-SMA, Vimentin, and PDGFR-*β* (Figure 2). As MSCs are found in almost all tissues of the human body, UdRPC-iMSCs are perfectly suited for the generation of organoids consisting of distinct cell types. MSCs have been described to be important for the process of self-condensation in the generation of organoids where contractions of the actomyosin cytoskeletal axis of MSCs play the key role [2, 26]. Condensation did not occur in the absence of MSCs and organoids could not form [2]. This observation was also made in this study; even though UdRPCs are MSCs, incubation of UdRPCs alone only led to emerging 3D cell aggregates without the typical round organoid structures with borders typical of 3D-NPCs (data not shown). It is known from embryonic invagination that Myosin II is active during this developmental process which leads to inward dislocation of cell-cell junctions [2, 26]. Takebe et al. were able to show that in MSCs, Myosin II was highly expressed just before condensation took place [2]. Furthermore, it has been shown that progressive recruitment of mesenchymal progenitors plays a fundamental role in cell fate acquisition during nephrogenesis in mice and human [27]. Another important role of MSCs was described by Tögel et al., where MSCs were injected into rat models suffering from reperfusion-induced acute renal failure [28]. The injected MSCs were able to protect renal cells from further damage and partly restored renal functions by secretion of anti-inflammatory factors.

### 3.2. Urine-Derived Renal Progenitor Cells (UdRPCs)

**Figure 3:**
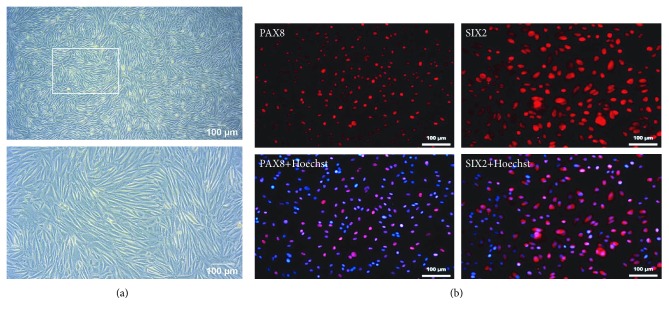
Phase contrast image of UdRPCs and expression of kidney-related markers. (a) Cell morphology of UdRPCs. (b) Stainings were carried out for PAX8 and the nephron progenitor marker SIX2. Cell nuclei were stained with Hoechst. Pictures were taken under 10x and 20x magnification.

UdRPCs were isolated from voided urine of a male donor of African origin [17]. When kept in proliferation medium, they retained the typical rice grain-like morphology (Figure 3(a)) and expressed PAX8 and SIX2 (Figure 3(b)).

### 3.3. Generation of Endothelial Cells from UdRPC-iPSCs

**Figure 4:**
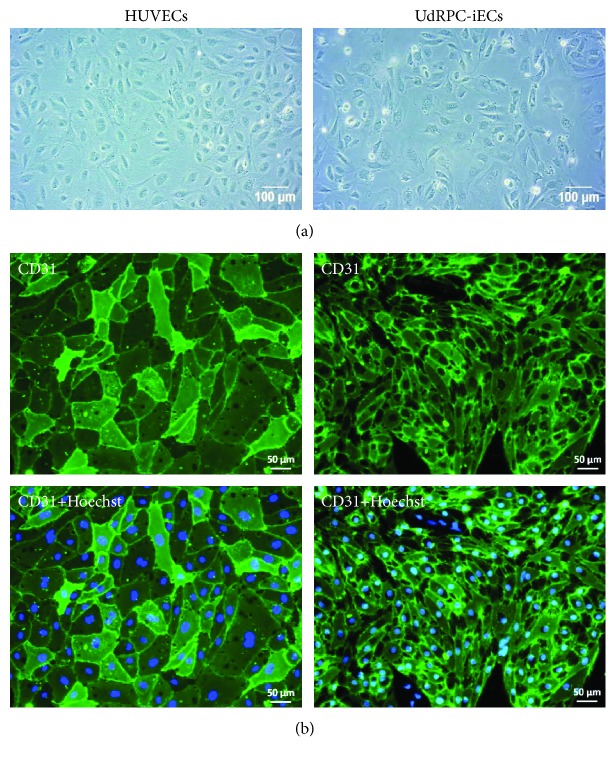
Comparison of UdRPC-iECs with HUVECs. (a) UdRPC-iECs had a broad, cobblestone-like morphology similar to HUVECs. (b) Expression of the endothelial cell surface marker CD31 in HUVECs and UdRPC-iECs. Cell nuclei were stained with Hoechst.

UdRPC-iPSCs were differentiated to endothelial cells (UdRPC-iECS) using a modified two-step protocol [18]. The differentiated cells had a cobblestone-like morphology with broad cell bodies and grew as a thin adherent cell layer (Figure 4(a)). Like HUVECs, UdRPC-iECs uniformly expressed the endothelial cell surface marker CD31 (Figure 4(b)). Cell sizes of UdRPC-iECs were smaller than those of HUVECs (Figure 4(b)) which could be explained by a lower passage number and the fact that they were derived from iPSCs which are small in size themselves. Since *in vivo* vasculature for nutrient and oxygen supply is established in the early embryonal development, UdRPC-iECs were used for the formation of kidney preorganoids which should support the sufficient availability with nutrients and oxygen and allow further maturation of kidney structures.

### 3.4. Formation of 3D-Nephron Progenitor Cells

**Figure 5:**
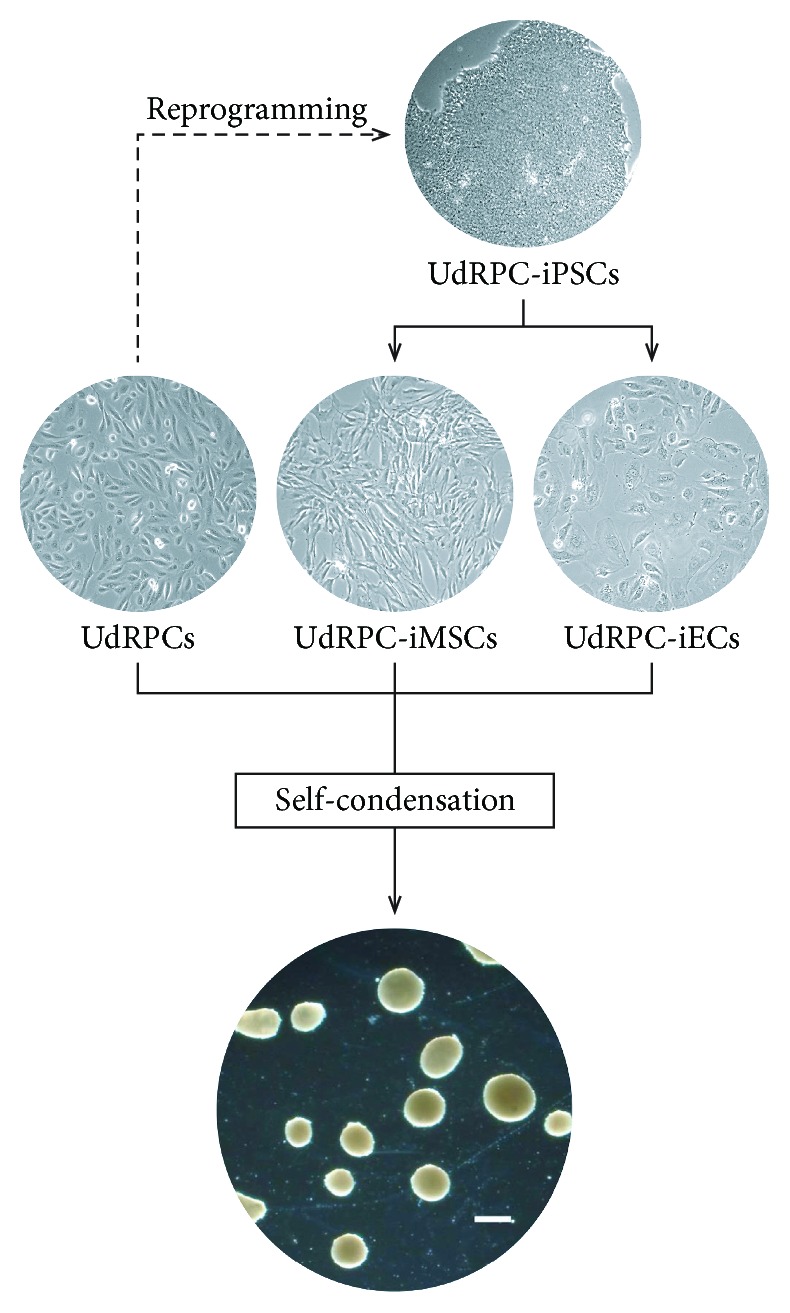
Overview of the formation of three-dimensional nephron progenitor cells (3D-NPCs). Isogenic 3D-NPCs were generated from three cell types namely SIX2-positive UdRPCs, UdRPC-iMSCs, and UdRPC-iECs of the same genetic background. The scale bar corresponds to a length of 500 *μ*m.

Three-dimensional nephron progenitor cells (3D-NPCs) were generated by combining urine-derived SIX2-positive renal progenitor cells (UdRPCs), UdRPC-iMSCs, and UdRPC-iECs at a ratio of 10 : 7 : 2. The cell mixture self-condensed after 2 to 4 days forming round-shaped, three-dimensional structures with sharp borders (Figure 5). The 3D-NPCs were transferred to petri dishes 14 days after the respective cells were combined.

### 3.5. Expression of Renal, Endothelial, and Mesenchymal Markers in 3D-NPCs

**Figure 6:**
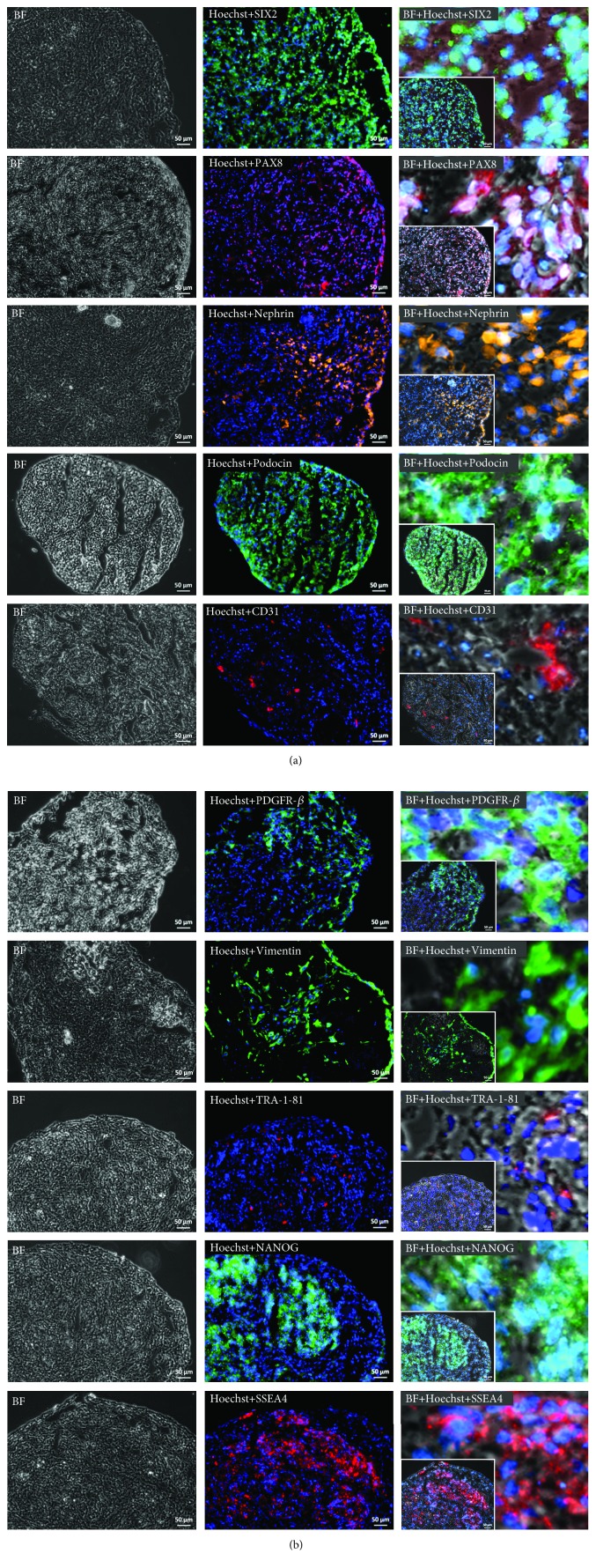
Expression of kidney markers (SIX2, PAX8, Nephrin, and Podocin), endothelial marker (CD31), mesenchymal markers (PDGFR-*β* and Vimentin), and pluripotency-associated markers (TRA-1-81, NANOG, and SSEA4) in 3D-NPCs. Cell nuclei were stained with Hoechst. Zoom-in pictures show the subcellular localization of the respective proteins. Pictures were taken under 20x magnification. The scale bars represent a length of 50 *μ*m.

After three to four weeks of cultivation, 3D-NPCs were fixed, dehydrated, and subsequently embedded in the preparation of cryosectioning. The sections were then stained for the expression of several kidney-specific markers, such as SIX2, PAX8, Nephrin, and Podocin, endothelial marker- CD31, and mesenchymal markers, PDGFR-*β* and Vimentin (Figure 6).

3D-NPCs express the renal progenitor marker SIX2 which in mice has been shown to be expressed during early kidney development, especially in the cap mesenchyme, a region consisting of progenitor cells committed to the nephron fate [27, 29]. This gene is involved in the maintenance of the progenitor state, and the depletion of SIX2 leads to the differentiation of the progenitor cells towards cell types making up the nephron, the functional unit of the kidney, including podocytes and distal and proximal tubules.

The early renal marker PAX8 is uniformly expressed in 3D-NPCs (Figure 6). PAX8 expression is maintained throughout nephron morphogenesis, emerging at the renal vesicle stage, and regulates kidney organogenesis [30, 31]. Cytoplasmic expression of Nephrin was not as uniform as seen for SIX2 and PAX8, but more localized (Figure 6). Nephrin is a protein of the immunoglobulin superfamily of cell adhesion receptors and is present in epithelial podocytes which wrap around the glomeruli and are part of the glomerular filtration barrier [32]. The podocytic foot processes are interconnected via slit diaphragms which are formed by Nephrin, Podocin, TRPC6, and FAT1 [33, 34]. Expression of the membrane protein Podocin, encoded by *NPHS2*, was detected on the plasma membrane of cells within the 3D-NPCs (Figure 6). It has to be noted that native UdRPCs express SIX2 [17], Nephrin, and Podocin (data not shown); therefore, it is further evidence in support of our generated 3D-NPCs.

Furthermore, 3D-NPCs harbour endothelial cells which express the cell surface marker CD31 (Figure 6). CD31 is also known as PECAM-1, a glycoprotein, and besides being present on the cell surface of endothelial cells, CD31 can also be found on platelets and some leukocytes [35]. This protein is involved in the adhesion between the endothelial cells by intercellular junctions [35]. Expression of the MSC markers Vimentin and PDGFR-*β* was not uniformly distributed as seen for PAX8. Vimentin is a type III intermediate filament, which forms the cytoskeleton together with microtubules and actin filaments. This protein is important for the maintenance of cell and tissue integrity [36]. Vimentin was also found to contribute to epithelial to mesenchymal transition (EMT) by upregulating the expression of EMT-related genes [37]. PDGFR-*β* is a receptor protein for the mitogen PDGF [38] and is involved in the development of mesenchymal stem cells. As mentioned before, MSCs are essential for self-condensation of organoids. In this case, UdRPC-iMSCs might have been involved in the condensation process where the contractile force of the cytoskeleton leads to 3D formation [2]. The addition of UdRPC-iMSCs should also be beneficial for the vascularization of 3D-NPCs. MSCs are in particular known to secrete a variety of growth factors and cytokines, some of them with proangiogenic properties such as VEGF-A, interleukin- (IL-) 6, IL-8, HGF, and PDGF [19, 38, 39].

Additionally, the sections were also stained for the expression of the pluripotency-associated proteins TRA-1-81, SSEA4, and NANOG (Figure 6). We chose to analyse NANOG expression because the cytoplasmic variant is known to be expressed in the kidney [40].

### 3.6. The Generation of 3D Kidney Organoids

The generation of kidney organoids has advanced in recent years. Compared to the 2D approach to cultivate renal tissues, 3D culture systems better mimic the *in vivo* configuration. Most protocols are based on the use of human pluripotent stem cells (ESCs and iPSCs) differentiated via formation of the intermediate mesoderm into renal structures [14, 41]. Alternatively, kidney tissues have been generated with a two-step protocol, starting with the formation of pluripotent stem cell-derived embryoid bodies followed by chemical-induced differentiation to kidney cell lineages including podocytes, cells of proximal and distal tubules, and collecting ducts [42]. In order to capture the complexity of the kidney organ, multicellular kidney spheroids from a coculture of PSCs, MSCs, and HUVECs driven by mesenchymal cell condensation were engineered by Takebe et al. [26] and Takahashi et al. [38]. Upon transplantation into mice, an *in vivo* environment, connection to the donor vasculature and self-organization into functional tissues fulfilling organ functions such as urine production were observed [26]. Moreover, instead of pluripotent stem cells, murine and human primary kidney cells isolated from biopsies have been described for the generation of three-dimensional renal structures *in vitro* [43, 44]. As renal development is completed before birth, isolation of human NPCs however is difficult. Several groups have worked on optimizing this isolation process as well as the *in vitro* cultivation conditions. Methods for the isolation of human NPCs from the human fetal kidney as well as long-term 3D culture of isolated fetal NPCs with retained nephrogenic potential have been described [45, 46]. With a similar nephrogenic potential as primary NPCs, our novel approach for the generation of 3D-NPCs was based on the use of UdRPCs in combination with isogenic UdRPC-iMSCs and UdRPC-iECs. As urine is an excretion product, isolation of UdRPCs is noninvasive, cost-effective, and indefinite [3]. Moreover, they can be isolated from every donor regardless of age, gender, and health condition. Additionally, even though these cells have moderate telomerase activity, they do not form teratomas or tumors [3, 5].

## 4. Conclusion

Summarizing our study, heterotypic 3D-NPCs were generated by combining UdRPCs, UdRPC-iPSC-derived UdRPC-iMSCs, and UdRPC-iECs originating from the same genetic background, hence isogenic. An immunofluorescence-based analysis demonstrated the expression of the renal progenitor markers (SIX2 and PAX8), the glomerular marker (Nephrin and Podocin), and the endothelial marker (CD31) as well as the mesenchymal markers (Vimentin and PDGFR-*β*). 3D-NPCs have renal progenitor characteristics and therefore have the potential to generate several cell types of the kidney lineage. As the 3D-NPCs arose from isogenic cell types, inducing the differentiation of renal cell types with subsequent organoid formation could lead to future use in cell replacement therapies, drug screening, and nephrotoxicity studies as well as kidney-associated disease modelling.

## Figures and Tables

**Table 1:** List of antibodies and dilution for immunofluorescence staining.

Immunofluorescence antibody	Specificity	Dilution	Company	Cat. No.
CD31	Mouse	1 : 300	R&D	BBA7
NPHS1	Rabbit	1 : 200	Invitrogen	PA5-20330
NPHS2	Rabbit	1 : 400	Proteintech	20384-1-AP
PAX8	Rabbit	1 : 200	Cell Signaling	59019
PDGFR-*β*	Rabbit	1 : 100	Cell Signaling	3169
SIX2	Rabbit	1 : 200	Proteintech	11562-1-AP
Vimentin	Rabbit	1 : 200	Cell Signaling	5741
Alexa 488	Rabbit	1 : 500	Invitrogen	A-11034
Alexa 555	Rabbit	1 : 500	Invitrogen	A-21428
Cy3	Mouse	1 : 500	Invitrogen	A10521
NANOG	Rabbit	1 : 800	Cell Signaling	4903T
SSEA4	Mouse	1 : 1000	Cell Signaling	4755T
TRA-1-81	Mouse	1 : 1000	Cell Signaling	4745T

## Data Availability

The photo and plot data used to support the findings of this study are included within the article and in the supplementary files.

## Supplementary Materials

Supplementary Materials Immunophenotype of fetal MSCs and native UdRPCs. Expression of MSC cell surface markers CD73, CD90, and CD105 and hematopoietic markers CD14, CD20, CD34, and CD45 was analysed. (A) Fetal MSCs. (B) Native UdRPCs. Histograms of IgG control are displayed in orange, and histograms of MSC markers are displayed in blue (*n* = 2).

## References

[1] Oliveira Arcolino F., Piella A. T., Papadimitriou E. (2015). Human urine as a noninvasive source of kidney cells. *Stem Cells International*.

[2] Takebe T., Zhang R. R., Koike H. (2013). Vascularized and functional human liver from an iPSC-derived organ bud transplant. *Nature*.

[3] Liu G., Zhang Y., Deng C., Turksen K. (2013). Urine-derived stem cells: biological characterization and potential clinical applications. *Stem Cells: Current Challenges and New Directions*.

[4] Bussolati B., Camussi G. (2015). Therapeutic use of human renal progenitor cells for kidney regeneration. *Nature Reviews Nephrology*.

[5] Zhang D., Wei G., Li P., Zhou X., Zhang Y. (2014). Urine-derived stem cells: a novel and versatile progenitor source for cell-based therapy and regenerative medicine. *Genes & Diseases*.

[6] Kobayashi A., Valerius M. T., Mugford J. W. (2008). Six2 defines and regulates a multipotent self-renewing nephron progenitor population throughout mammalian kidney development. *Cell Stem Cell*.

[7] Bartfeld S., Clevers H. (2017). Stem cell-derived organoids and their application for medical research and patient treatment. *Journal of Molecular Medicine*.

[8] Fatehullah A., Tan S. H., Barker N. (2016). Organoids as an in vitro model of human development and disease. *Nature Cell Biology*.

[9] Simunovic M., Brivanlou A. (2017). Embryoids, organoids and gastruloids: new approaches to understanding embryogenesis. *Development*.

[10] Eiraku M., Takata N., Ishibashi H. (2011). Self-organizing optic-cup morphogenesis in three-dimensional culture. *Nature*.

[11] Suga H., Kadoshima T., Minaguchi M. (2011). Self-formation of functional adenohypophysis in three-dimensional culture. *Nature*.

[12] Sato T., Clevers H. (2013). Growing self-organizing mini-guts from a single intestinal stem cell: mechanism and applications. *Science*.

[13] Lancaster M. A., Knoblich J. A. (2014). Organogenesis in a dish: modeling development and disease using organoid technologies. *Science*.

[14] Takasato M., Er P. X., Chiu H. S., Little M. H. (2016). Generation of kidney organoids from human pluripotent stem cells. *Nature Protocols*.

[15] Takebe T., Zhang R. R., Koike H. (2014). Generation of a vascularized and functional human liver from an iPSC-derived organ bud transplant. *Nature Protocols*.

[16] Zhou T., Benda C., Duzinger S. (2012). Generation of human induced pluripotent stem cells from urine samples. *Nature Protocols*.

[17] Bohndorf M., Ncube A., Spitzhorn L.-S., Enczmann J., Wruck W., Adjaye J. (2017). Derivation and characterization of integration-free iPSC line ISRM-UM51 derived from SIX2-positive renal cells isolated from urine of an African male expressing the CYP2D6 ∗4/∗17 variant which confers intermediate drug metabolizing activity. *Stem Cell Research*.

[18] Zhang J., Schwartz M. P., Hou Z. (2017). A genome-wide analysis of human pluripotent stem cell-derived endothelial cells in 2D or 3D culture. *Stem Cell Reports*.

[19] Spitzhorn L.-S., Kordes C., Megges M. (2018). Transplanted human pluripotent stem cell-derived mesenchymal stem cells support liver regeneration in Gunn rats. *Stem Cells and Development*.

[20] Chen Y. S., Pelekanos R. A., Ellis R. L., Horne R., Wolvetang E. J., Fisk N. M. (2012). Small molecule mesengenic induction of human induced pluripotent stem cells to generate mesenchymal stem/stromal cells. *Stem Cells Translational Medicine*.

[21] Mirmalek-Sani S.-H., Tare R. S., Morgan S. M. (2009). Characterization and multipotentiality of human fetal femur-derived cells: implications for skeletal tissue regeneration. *Stem Cells*.

[22] Dominici M., Le Blanc K., Mueller I. (2006). Minimal criteria for defining multipotent mesenchymal stromal cells. The International Society for Cellular Therapy position statement. *Cytotherapy*.

[23] Hass R., Kasper C., Böhm S., Jacobs R. (2011). Different populations and sources of human mesenchymal stem cells (MSC): a comparison of adult and neonatal tissue-derived MSC. *Cell Communication and Signaling*.

[24] Bharadwaj S., Liu G., Shi Y. (2013). Multipotential differentiation of human urine-derived stem cells: potential for therapeutic applications in urology. *Stem Cells*.

[25] Lang R., Liu G., Shi Y. (2013). Self-renewal and differentiation capacity of urine-derived stem cells after urine preservation for 24 hours. *PLoS One*.

[26] Takebe T., Enomura M., Yoshizawa E. (2015). Vascularized and complex organ buds from diverse tissues via mesenchymal cell-driven condensation. *Cell Stem Cell*.

[27] Lindström N. O., De Sena G., Tran T. (2018). Progressive recruitment of mesenchymal progenitors reveals a time-dependent process of cell fate acquisition in mouse and human nephrogenesis. *Developmental Cell*.

[28] Tögel F., Hu Z., Weiss K., Isaac J., Lange C., Westenfelder C. (2005). Administered mesenchymal stem cells protect against ischemic acute renal failure through differentiation-independent mechanisms. *American Journal of Physiology Renal Physiology*.

[29] Self M., Lagutin O. V., Bowling B. (2006). Six2 is required for suppression of nephrogenesis and progenitor renewal in the developing kidney. *The EMBO Journal*.

[30] Little M. H., McMahon A. P. (2012). Mammalian kidney development: principles, progress, and projections. *Cold Spring Harbor Perspectives in Biology*.

[31] Barr M. L., Jilaveanu L. B., Camp R. L., Adeniran A. J., Kluger H. M., Shuch B. (2015). PAX-8 expression in renal tumours and distant sites: a useful marker of primary and metastatic renal cell carcinoma?. *Journal of Clinical Pathology*.

[32] Narlis M., Grote D., Gaitan Y., Boualia S. K., Bouchard M. (2007). *Pax2* and *Pax8* regulate branching morphogenesis and nephron differentiation in the developing kidney. *Journal of the American Society of Nephrology*.

[33] Ristola M., Lehtonen S. (2014). Functions of the podocyte proteins nephrin and Neph3 and the transcriptional regulation of their genes. *Clinical Science*.

[34] Rabelink T. J., Heerspink H. J. L., Zeeuw D., Rosenberg P. L. K. M. (2015). Chapter 9 - the pathophysiology of proteinuria. *Chronic Renal Disease*.

[35] Weber S., Geary D. F., Schaefer F. B. T.-C. P. N. (2008). Chapter 13 - hereditary nephrotic syndrome. *Comprehensive Pediatric Nephrology*.

[36] Chuah J. K. C., Zink D. (2017). Stem cell-derived kidney cells and organoids: recent breakthroughs and emerging applications. *Biotechnology Advances*.

[37] Liu C.-Y., Lin H.-H., Tang M.-J., Wang Y.-K. (2015). Vimentin contributes to epithelial-mesenchymal transition cancer cell mechanics by mediating cytoskeletal organization and focal adhesion maturation. *Oncotarget*.

[38] Takahashi Y., Sekine K., Kin T., Takebe T., Taniguchi H. (2018). Self-condensation culture enables vascularization of tissue fragments for efficient therapeutic transplantation. *Cell Reports*.

[39] Rahman M. S., Spitzhorn L.-S., Wruck W. (2018). The presence of human mesenchymal stem cells of renal origin in amniotic fluid increases with gestational time. *Stem Cell Research & Therapy*.

[40] Das S., Jena S., Levasseur D. N. (2011). Alternative splicing produces Nanog protein variants with different capacities for self-renewal and pluripotency in embryonic stem cells. *Journal of Biological Chemistry*.

[41] Takasato M., Er P. X., Becroft M. (2014). Directing human embryonic stem cell differentiation towards a renal lineage generates a self-organizing kidney. *Nature Cell Biology*.

[42] Przepiorski A., Sander V., Tran T. (2018). A simple bioreactor-based method to generate kidney organoids from pluripotent stem cells. *Stem Cell Reports*.

[43] Joraku A., Stern K. A., Atala A., Yoo J. J. (2009). In vitro generation of three-dimensional renal structures. *Methods*.

[44] Guimaraes-Souza N. K., Yamaleyeva L. M., AbouShwareb T., Atala A., Yoo J. J. (2012). In vitro reconstitution of human kidney structures for renal cell therapy. *Nephrology Dialysis Transplantation*.

[45] Da Sacco S., Thornton M. E., Petrosyan A. (2016). Direct isolation and characterization of human nephron progenitors. *Stem Cells Translational Medicine*.

[46] Li Z., Araoka T., Wu J. (2016). 3D culture supports long-term expansion of mouse and human nephrogenic progenitors. *Cell Stem Cell*.

