# Supplementary figures and images for: Constructing an Isogenic 3D Human Nephrogenic Progenitor Cell Model Composed of Endothelial, Mesenchymal, and SIX2-Positive Renal Progenitor Cells
Source: Stem Cells Int. 2019 May 2;2019:3298432. doi: 10.1155/2019/3298432 (PMC6525793; doi:10.1155/2019/3298432)

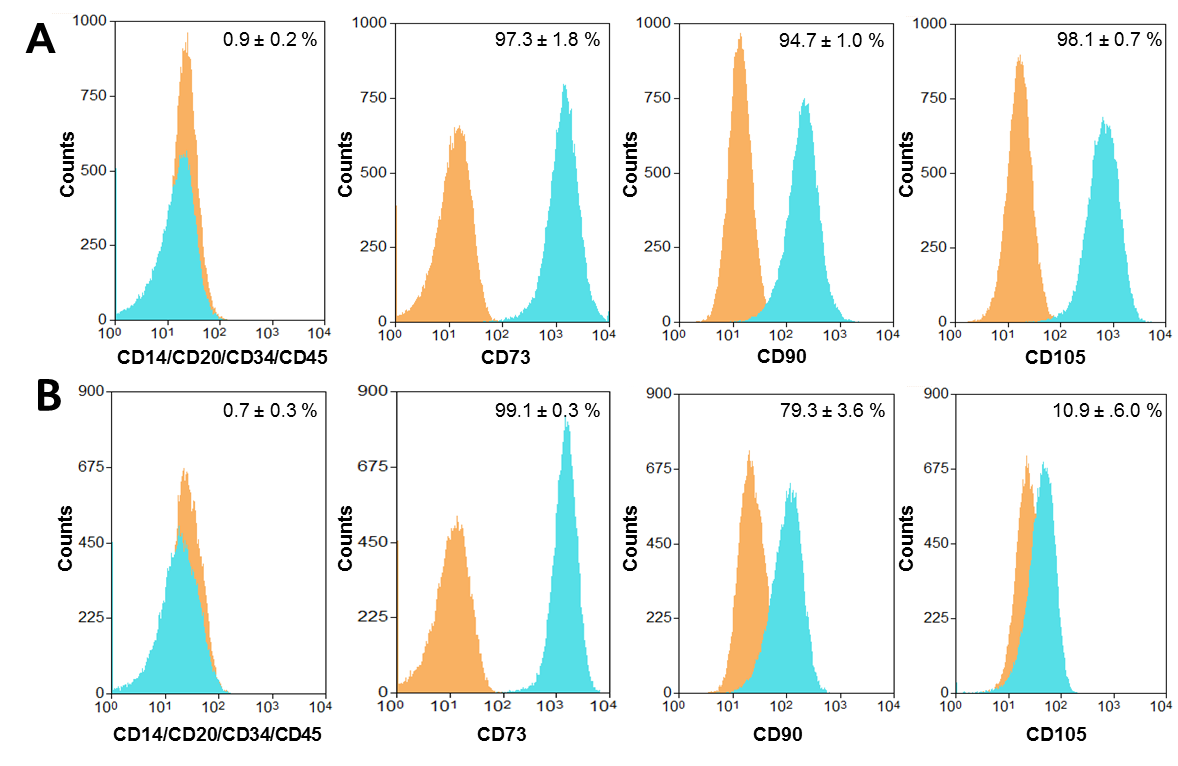

Supplement: Supplementary Materials — Immunophenotype of fetal MSCs and native UdRPCs. Expression of MSC cell surface markers CD73, CD90, and CD105 and hematopoietic markers CD14, CD20, CD34, and CD45 was analysed. (A) Fetal MSCs. (B) Native UdRPCs. Histograms of IgG control are displayed in orange, and histograms of MSC markers are displayed in blue (n = 2). [file 3298432.f1.tif]
